# The prognostic value of weight loss during radiotherapy among patients with nasopharyngeal carcinoma: a large-scale cohort study

**DOI:** 10.1186/s12885-022-09562-9

**Published:** 2022-05-06

**Authors:** Ya-Nan Jin, Tian-Liang Xia, Dong-Mei Mai, Ji-Jin Yao, Chang Jiang, Wen-Zhuo He, Liang-Ping Xia

**Affiliations:** 1 VIP Region, Sun Yat-sen University Cancer Center, State Key Laboratory of Oncology in South China, Guangdong Key Laboratory of Nasopharyngeal Carcinoma Diagnosis and Therapy, Collaborative Innovation Center for Cancer Medicine, Guangzhou, 510060 Guangdong China; 2 The Cancer Center of the Fifth Affiliated Hospital of Sun Yat-sen University, Guangdong Provincial Key Laboratory of Biomedical Imaging, Zhuhai, 510060 Guangdong China; 3 State Key Laboratory of Oncology in South China, Collaborative Innovation Center for Cancer Medicine, Guangdong Key Laboratory of Nasopharyngeal Carcinoma Diagnosis and Therapy, Sun Yat-sen University Cancer Center, Guangzhou, 510060 Guangdong China; 4 Department of Anaesthesiology, Sun Yat-sen University Cancer Center, State Key Laboratory of Oncology in South China, Collaborative Innovation Center for Cancer Medicine, Guangdong Key Laboratory of Nasopharyngeal Carcinoma Diagnosis and Therapy, Guangzhou, 510060 Guangdong China

**Keywords:** Nasopharyngeal carcinoma, Radiotherapy, Weight loss, Survival, Prognostic value

## Abstract

**Background:**

We aim to investigate the prognostic value of weight loss during radiotherapy (RT) among patients with nasopharyngeal carcinoma (NPC).

**Methods:**

A total of 1149 NPC patients who received radical RT were retrospectively analyzed. Patients’ weight were measured at initiation of RT (W_Pre-RT_) and every week during RT (W_RT1,2,3,4,5,6,7_). Percentage of weight loss (PWL) at 1st, 2nd, 3rd, 4th, 5th, 6th, and 7th week of RT (RT-PWL_1,2,3,4,5,6,7_) were calculated using the following equation: (W_Pre-RT_ –W_RT1,2,3,4,5,6,7_)/W_Pre-RT_ × 100%. The optimal threshold of RT-PWL_7_ was determined by recursive partitioning analyses (RPAs). Our endpoints included disease-free survival (DFS), overall survival (OS), distant metastasis-free survival (DMFS), and locoregional relapse-free survival (LRRFS).

**Results:**

The median RT-PWLs were 0, 0, 1.5, 2.9, 4.1, 5.5, 6.6% at 1st, 2nd, 3rd, 4th, 5th, 6th, and 7th week of RT, respectively. RT-PWL_7_ optimal threshold with respect to DFS was 5.3% based on RPAs. Therefore, a consistent threshold of 5% (<5% vs > ≥5%) was selected to classify NPC patients into low RT-PWL_7_ and high RT-PWL_7_ groups for survival analysis. Compared to high RT-PWL_7_ (≥5%), patients with low RT-PWL_7_ (< 5%) had significantly better ten-year DFS (61.2% vs 78.8%; *P* < 0.001), OS (70.1% vs 86.6%; P < 0.001), and DMFS (80.2% vs 88.5%; *P* = 0.007). However, no difference was observed between LRRFS groups (91.7% vs 94.3%; *P* = 0.173). In multivariate analysis, high RT-PWL_7_ was an independent risk factor for DFS (HR, 1.56; 95%CI, 1.19-2.03; *P* = 0.001), OS (HR, 1.54; 95%CI, 1.11-2.15; *P* = 0.011), and DMFS (HR, 1.47; 95%CI, 1.03-2.10; *P* = 0.033) in patients with NPC. In addition, treatment strategy, plasma Epstein-Barr virus DNA, and N stage were associated with weight loss.

**Conclusions:**

High RT-PWL_7_ was significantly associated with decreased DFS, OS, and DMFS for NPC patients. Clinicians should continuously inform patients on the health impact of minimizing RT-PWL_7_ under 5% during radiotherapy.

## Background

Nasopharyngeal carcinoma (NPC), an epithelial malignancy that is distinguished from other head and neck cancers, is highly prevalent in southern China [1]. The main treatment for NPC is radiotherapy (RT) due to anatomical restrictions and radio-sensitivity. Over the past decade, advances in imagining techniques, chemotherapy, and radiation technology contributed to improved NPC survival. However, 20–30% of patients still die because of NPC recurrence [2, 3]. Therefore, efforts to identify modifiable risk factors can potentially provide new insights on developing clinical intervention for increasing long-term survival.

Patients diagnosed with head and neck cancers often experience weight loss during RT due to acute toxicity, such as mucositis, dysgeusia, xerostomia, and nausea [4–7]. Previous studies [8, 9] have estimated the incidences of weight loss to range from 40 to 90%, especially among NPC patients where rates were high. Substantial weight loss during treatment was significantly associated with poor survival among NPC patients [10–13]. Monitoring decreasing weight during RT will allow for clinicians to evaluate the current treatment plan effectiveness for NPC [14]. Knowing the influencing factors for weight loss during RT is helpful in selecting patients for preventive measures before RT and altering RT treatment. To date, prior studies only captured bodyweight at baseline visit and again at the end of treatment [10–12], without considering the downward trend of weight loss during RT period.

To fill current gaps in knowledge, we conducted a large-scale retrospective study of NPC patients treated with radical RT. The present study sought to (1) draw a downward trend of weight loss during RT; (2) identify the weight loss prognostic value on survival outcomes; and (3) demonstrate risk factors for weight loss among NPC patients.

## Methods

### Patient characteristics

The present study was a retrospective cohort study utilizing an NPC-specific database from Sun Yat-Sen University Cancer Center between January 2006 and October 2014. We included patients if they met the following criteria: (1) newly diagnosed non-disseminated NPC; (2) Karnosfky performance score (KPS) ≥ 80; (3) no indication of distant metastases; (4) absent of secondary malignancy; (5) treated with radical intensity-modulated radiotherapy (IMRT); and (6) complete bodyweight information. This study was approved by the Institutional Review Board of Sun Yat-Sen University Cancer Center, and informed consent was obtained from all patients.

A total of 1149 patients were included in our study. The baseline assessment included full physical examination, fiberoptic nasopharyngoscopy, magnetic resonance imaging, computed tomography, abdominal ultrasonography, biochemistry profiling and hematology, whole body bone scan or ^18^F-fluorodeoxyglucose positron emission tomography and computed tomography. Real-time quantitative polymerase chain reaction was used to measure Epstein-Barr virus (EBV) DNA concentrations as previously described in detail [15]. Patients were staged based on the 7th edition of the American Joint Commission on Cancer (AJCC) staging system [16].

### Radiotherapy and chemotherapy

All patients received radical IMRT in the current study. Dose prescribed to patients were (1) 66-70 Gy at 2.12-2.27 Gy/fraction to planning target volume (PTV) of nasopharyngeal gross tumor volume (GTVnx); (2) PTV of GTV of the metastatic lymph nodes (GTVnd) received 64-70 Gy; (3) high-risk clinical target volume (CTV1) received 60-63 Gy to PTV; and (4) low-risk clinical target volume (CTV2) received 50-56 Gy to PTV. During the study period, institutional guidelines recommended no chemotherapy for patients with stage I, and concurrent chemoradiotherapy +/− neoadjuvant/adjuvant chemotherapy for stages II to IVB, as defined by the 7th edition of AJCC staging system. Neoadjuvant or adjuvant chemotherapy consisted of cisplatin (60 mg/m^2^), docetaxel (60 mg/m^2^), and 5-fluorouracil (600 mg/m^2^/day over 120 h), or cisplatin (80 mg/m^2^) plus 5-fluorouracil (800 mg/m^2^/day over 120 h) or cisplatin (80 mg/m^2^) plus docetaxel (80 mg/m^2^) every 3 weeks for three cycles. Concurrent chemotherapy comprised of cisplatin (80 or 100 mg/m^2^) given in weeks one, four, and seven of RT, or cisplatin (40 mg/m^2^) given weekly during radiotherapy.

### Data collection

Patients’ age, height, weight, sex, pre-therapy laboratory counts of serum lactate dehydrogenase (LDH), high sensitivity C-reactive protein (hs-CRP), plasma EBV DNA, pathological types, clinical stage, and treatment type were extracted from medical records. Digital electronic scale (XiangShan, EB9871) was used to measure bodyweight to the nearest 0.1 kg in light garment and without shoes. We measured patients’ bodyweight at initiation of RT and every week during RT. Bodyweight before RT (W_Pre-RT_) was measured at initiation of RT, and W_RT1,2,3,4,5,6,7_ (body weight at 1st, 2nd, 3rd, 4th, 5th, 6th, and 7th week of RT) was measured at each week of RT. The RT-PWL_1,2,3,4,5,6,7_ (percentage of weight loss at week 1, 2, 3, 4, 5, 6, 7 of RT) was calculated using the following equation: (W_Pre-RT_ –WRT_1,2,3,4,5,6,7_)/W_Pre-RT_ × 100%. 

Bodyweight before NAC (W_Pre-NAC_) was also measured at initiation of NAC for patients who received NAC before RT. NAC-PWL was calculated using the following equation: (W_Pre-NAC_ – W_Pre-RT)_/W_Pre-NAC_ × 100%.

At time of study, all patients were on 100% oral intake, where no type of enteral feeding tube or total parental nutrition were used.

### Follow-up and endpoints

Patients were examined every 3 months during the first 2 years, and every 6 months for years three through five, and annually thereafter until death. Disease-free survival (DFS) was our primary endpoint, defined as time from treatment diagnos to documented recurrence of disease (either distant metastasis or locoregional disease recurrence) or mortality from any cause, whichever occurred first. Secondary endpoints consisted of (1) distant metastasis free survival (DMFS) (no documented distant metastasis); (2) locoregional relapse free survival (LRRFS) (no documented locoregional recurrence); and (3) overall survival (OS).

### Statistical methods

In this study, we dichotomized the RT-PWL_7_ (percentage of weight loss at week 7 of RT) based on the optimal threshold: a RT-PWL_7_ of 5%, which was identified using the recursive partitioning analysis (RPA). Other variables such as host factors (e.g. age, gender, smoking history, hs-CRP, LDH, and plasma EBV DNA), treatment factors (e.g. treatment modality), and tumor factors (e.g. histology type, T stage, and N stage) were also grouped according to cutoff points from prior findings [17–19]. We first used the Kaplan-Meier method followed by the log-rank test to display the survival rate by the follow-up time and compare the difference in survival rates between the RT-PWL_7_ <  5% group and the RT-PWL_7_ ≥ 5% group. For each time-to-event outcome, we then developed univariate COX regression models to evaluate the association between the outcome and each of the independent variables, and included those with a *P* < 0.1 into the multivariate COX regression model. Hazard ratios (HRs) from the multivariate COX regression models were reported to describe the potential impact of RT-PWL_7_ after controlling for confounders. Furthermore, we investigated the potential factors associated with RT-PWL_7_ using logistic regression models. All statistical tests and *p*-values were two-sided. Analyses were conducted in R version 4.1.0 (http://www.r-project.org/).

## Results

### Patient characteristics

Clinicopathological characteristics of the 1149 patients are shown in Table 1. The median age was 45 years (range, 10–78 years), and the male/female ratio was 3.5:1. The percentage of patients at stage I, II, III, and IVA-B were 2.1, 9.9, 53.7, and 34.3%, respectively. During treatment, 140 patients (12%) received no chemotherapy, 549 (48%) received NAC, and 1009 (88%) received concurrent chemotherapy. Only 48% (262/549) patients experienced weight loss during NAC, but up to 92% (1058/1149) of patients experienced weight loss during RT. The median follow-up time was 72.6 months (interquartile range [IQR], 54.6–85.8 months).

**Table 1:** Baseline patient characteristics according to rate of weight loss during radiotherapy

Characteristic	Total (*N* = 1149)^a^	No. (%) of patients by RT-PWL		*P* value
		< 5% (*n* = 402, 35%)	≥5% (*n* = 747, 65%)	
Gender				0.826
Male	892 (77.6)	314 (78.1)	578 (77.4)	
Female	257 (22.4)	88 (21.2)	169 (22.6)	
Age, years				0.665
≤ 45	594 (51.7)	204 (50.7)	390 (52.2)	
> 45	555 (48.3)	198 (49.3)	357 (47.8)	
Histology (WHO)				0.732
Type I-II	35 (3.1)	11 (2.7)	24 (3.2)	
Type III	1114 (96.9)	391 (97.3)	723 (96.8)	
Smoking history				0.996
No	807 (70.2)	282 (70.2)	525 (70.3)	
Yes	342 (29.8)	120 (29.9)	222 (29.7)	
T stage (7th edition)				0.031
T1	71 (6.2)	32 (8.0)	39 (5.2)	
T2	185 (16.1)	69 (17.2)	116 (15.5)	
T3	627 (54.6)	226 (56.2)	401 (53.7)	
T4	266 (23.2)	75 (18.7)	191 (25.6)	
N stage (7th edition)				< 0.001
N0	128 (11.1)	59 (14.7)	69 (9.2)	
N1	448 (39.0)	177 (44.0)	271 (36.3)	
N2	399 (34.7)	118 (29.4)	281 (37.6)	
N3	174 (15.1)	48 (11.9)	126 (16.9)	
Overall stage				0.001
Stage I	24 (2.1)	13 (3.2)	11 (1.5)	
Stage II	114 (9.9)	53 (13.2)	61 (8.2)	
Stage III	617 (53.7)	223 (55.5)	394 (52.7)	
Stage IVA-B	394 (34.3)	113 (28.1)	281 (37.6)	
hs-CRP, g/mL^b^				0.536
< 1.0	363 (31.6)	133 (33.1)	230 (30.8)	
1.0-3.0	370 (32.2)	132 (32.8)	238 (31.9)	
≥ 3.0	416 (36.2)	137 (34.1)	279 (37.4)	
LDH, U/L^b^				0.095
< 245	1058 (92.1)	378 (94.0)	680 (91.0)	
≥ 245	91 (7.9)	24 (6.0)	67 (9.0)	
EBV DNA, copy/mL^b^				0.006
< 4000	766 (66.7)	289 (71.9)	477 (63.9)	
≥ 4000	383 (33.3)	113 (28.1)	270 (36.1)	
Treatment strategy				< 0.001
RT alone	140 (12.2)	81 (20.2)	59 (7.9)	
CCRT alone	460 (40.0)	126 (31.3)	334 (44.7)	
NAC + CCRT	549 (47.8)	195 (48.5)	354 (47.4)	

*Abbreviations*: *RT-PWL* Percentage of weight loss during radiotherapy, *hs-CRP* High sensitivity C-reactive protein, *LDH* Lactate dehydrogenase, *EBV* Epstein-Barr virus, *RT* Radiotherapy, *CCRT* Concurrent chemoradiotherapy, *NAC* Neoadjuvant chemotherapy

^a^ Percentages may not add up to 100 due to rounding

^b^ All variables were measured before treatment

### Variation of bodyweight loss during treatment

**Fig. 1:**
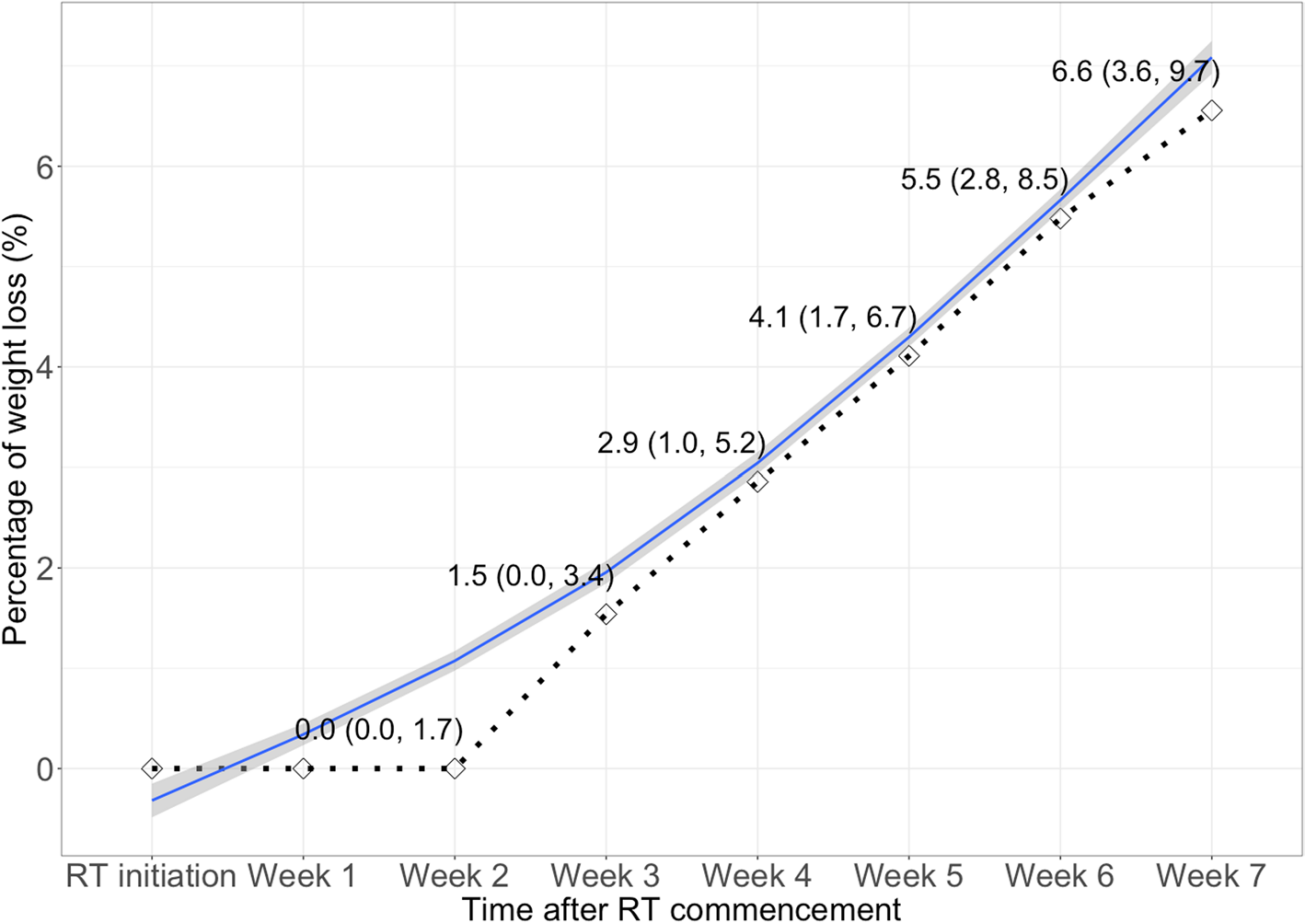
Downward trends in weight loss at every week during radiotherapy among patients with nasopharyngeal carcinoma

The median weight loss during NAC was 0.5 kg (IQR, 0 to 2.0 kg), and the median NAC-PWL was 1.1% (IQR, 0 to 3.3%). In contrast, the median weight loss during RT was 4.0 kg (IQR, 2.0 to 6.0 kg) and the median RT-PWL_7_ was 6.6% (IQR, 3.6 to 9.7%). We further outlined the downward trends of weight loss in the Fig. 1. Our results indicated RT-PWL remained largely unchanged in the first 2 weeks of RT, and then began to drop continuously at the following 5 weeks of RT (from 0 to 6.6%; at a percentage of about 1.3% weight loss per week).

### Prognostic value of bodyweight loss in patients with NPC

**Fig. 2:**
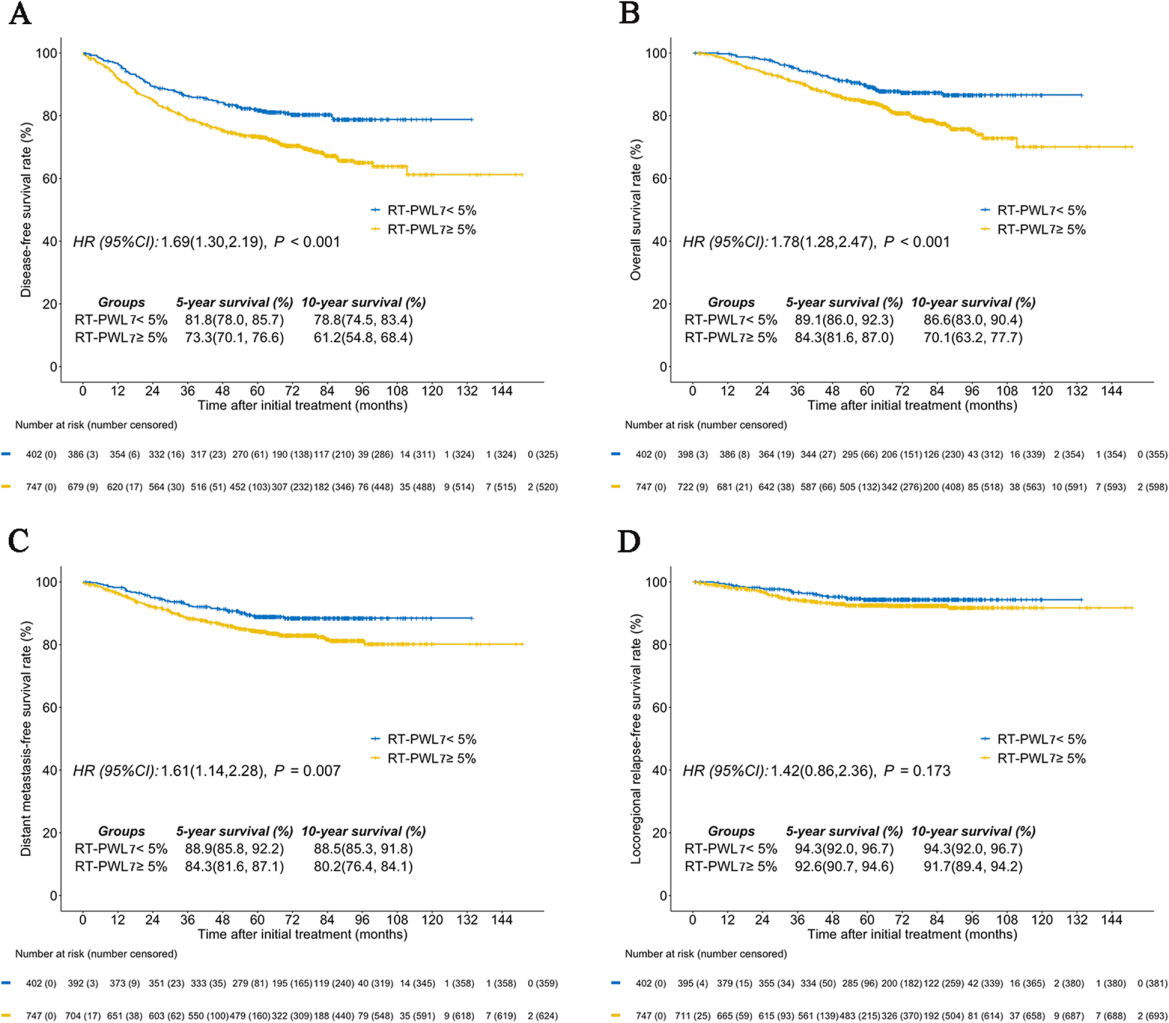
Comparison between the RT-PWL_7_ < 5% group and the RT-PWL_7_ ≥ 5% group for **A** disease-free survival, **B** overall survival, **C** distant metastasis-free survival, and **D** locoregional relapse-free survival. RT-PWL_7_, percentage of weight loss at week 7 of radiotherapy (RT)

**Fig. 3:**
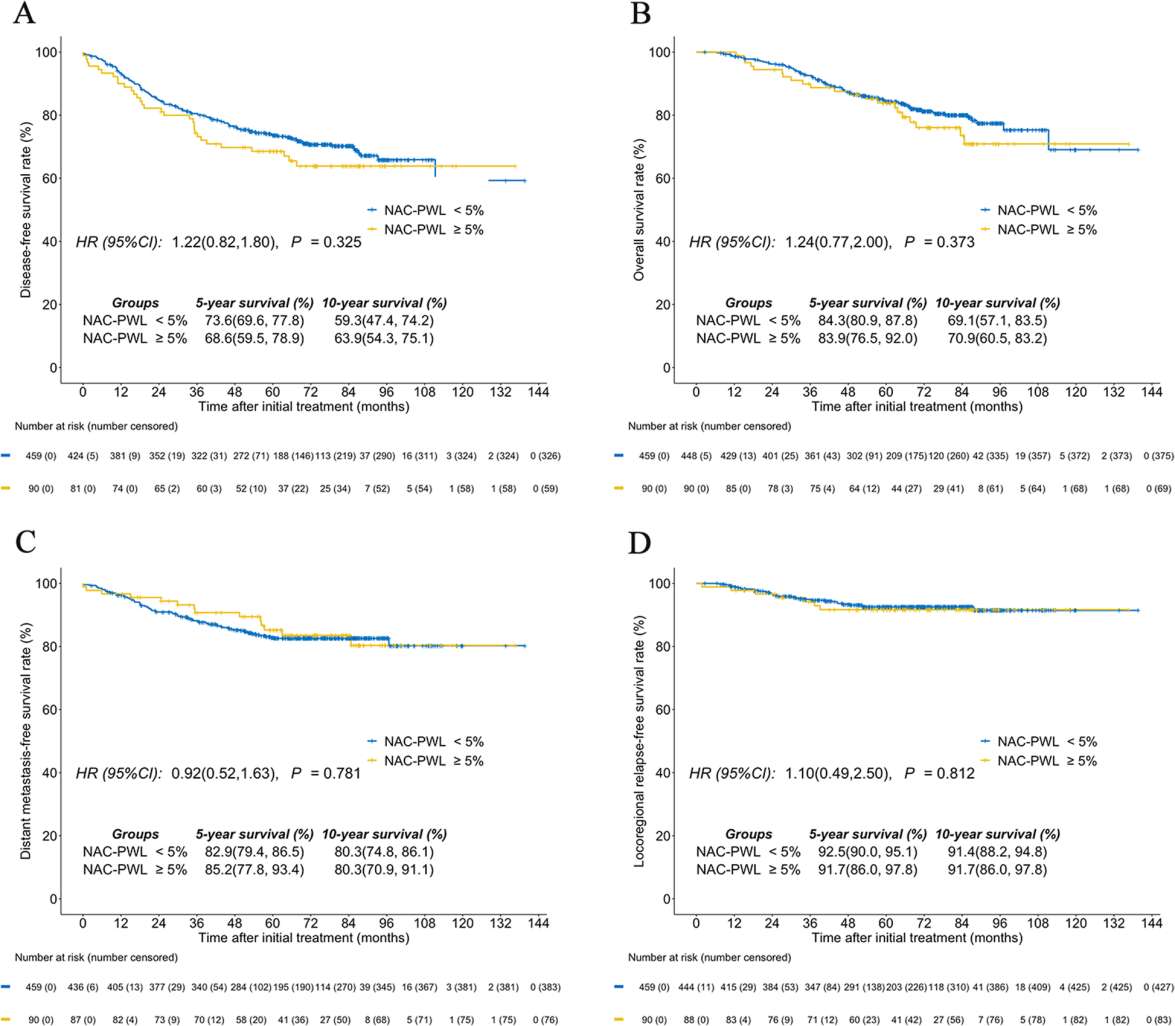
Comparison between the NAC-PWL < 5% group and the NAC-PWL ≥ 5% group for **A** disease-free survival, **B** overall survival, **C** distant metastasis-free survival, and **D** locoregional relapse-free survival. NAC-PWL: percentage of weight loss during neoadjuvant chemotherapy (NAC)

Five- and ten-year DFS, OS, DMFS, and LRRFS rates were 76.3 and 66.8%, 86.0 and 75.3%, 85.9 and 83.1%, and 93.2 and 92.7%, respectively. For the RT-PWL_7_, the optimal cutoff point for DFS among the entire group was 5.3% based on RPAs. Thus, a uniform cutoff point was selected at 5% (< 5% vs ≥5%) to classify patients into groups low RT-PWL_7_ and high RT-PWL_7_ for survival analysis. Overall, 65% (747/1149) of patients suffered ≥5% weight loss. When comparing survival between groups, our findings showed the high RT-PWL_7_ group had poorer ten-year DFS (61.2% vs. 78.8%; *P* < 0.001; Fig. 2A), OS (70.1% vs. 86.6%; *P* < 0.001; Fig. 2B), and DMFS (80.2% vs. 88.5%; *P* = 0.007; Fig. 2C) compared to low RT-PWL_7_ patients. No associated difference between groups for ten-year LRRFS (91.7% vs. 94.3%; *P* = 0.173; Fig. 2D) was observed. In multivariate analyses, RT-PWL_7_ ≥ 5% was an independent unfavorable prognostic factor for DFS (HR, 1.56; 95%CI, 1.19-2.03; *P* = 0.001), OS (HR, 1.54; 95%CI, 1.11-2.15; *P* = 0.011), and DMFS (HR, 1.47; 95%CI, 1.03-2.10; *P* = 0.033) (Table 2). The prognostic value of NAC-PWL for patients that received NAC was assessed as well. Consistent with RT-PWL_7_, a uniform cutoff point of 5% (< 5% versus ≥5%) was selected for survival analysis. In contrast, the ten-year rates of DFS (59.3% vs. 63.9%; *P* = 0.325; Fig. 3A), DMFS (69.1% vs. 70.9%; *P* = 0.373; Fig. 3B), OS (80.3% vs. 80.3%; *P* = 0.781; Fig. 3C), and LRFS (91.4% vs. 91.7%; *P* = 0.812; Fig. 3D) were comparable between patients who experienced NAC-PWL <  5% and those who experienced NAC-PWL ≥ 5%.

**Table 2:** Multivariate analysis of prognostic factors for patients with NPC (*n* = 1149)

Endpoint	Variable	HR	95% CI for HR	*P*^*a*^
DFS	RT-PWL (< 5% vs ≥ 5%)	1.56	1.19–2.03	0.001
	Age (≤ 45 vs > 45, years)	1.36	1.08–1.71	0.010
	Gender (Male vs Female)	1.18	0.91–1.53	0.215
	Pathology (Type I-II vs Type III)	0.44	0.26–0.72	0.001
	T stage (T1-2 vs T3-4)	1.62	1.16–2.27	0.005
	N stage (N0-1 vs N2-3)	1.54	1.20–1.97	0.001
	EBV DNA (< 4000 vs ≥ 4000, copy/mL)	1.50	1.12–2.01	0.007
	Treatment strategy (RT alone vs CCRT)	0.75	0.48–1.17	0.200
	Treatment strategy (RT alone vs NAC + CCRT)	0.84	0.54–1.32	0.455
OS	RT-PWL (< 5% vs ≥ 5%)	1.54	1.11–2.15	0.011
	Age (≤ 45 vs > 45, years)	1.57	1.17–2.10	0.002
	Pathology (Type I-II vs Type III)	0.37	0.21–0.66	0.001
	T stage (T1-2 vs T3-4)	1.84	1.20–2.81	0.005
	N stage (N0-1 vs N2-3)	1.72	1.26–2.81	0.001
	Smoking history (No vs Yes)	1.26	0.93–1.70	0.131
	LDH (< 245 vs ≥245, U/L)	1.27	0.80–2.03	0.308
	EBV DNA (< 4000 vs ≥ 4000, copy/mL)	1.54	1.08–2.21	0.018
DMFS	RT-PWL (< 5% vs ≥ 5%)	1.47	1.03–2.10	0.033
	Age (≤ 45 vs > 45, years)	1.43	1.04–1.96	0.026
	Pathology (Type I-II vs Type III)	0.55	0.27–1.13	0.104
	T stage (T1-2 vs T3-4)	1.41	0.92–2.18	0.118
	N stage (N0-1 vs N2-3)	1.99	1.41–2.80	< 0.001
	Smoking history (No vs Yes)	1.37	0.99–1.90	0.055
	LDH (< 245 vs ≥245, U/L)	1.33	0.82–2.16	0.247
	EBV DNA (< 4000 vs ≥ 4000, copy/mL)	2.01	1.23–3.30	0.006
LRRFS	Pathology (Type I-II vs Type III)	0.31	0.14–0.72	0.006
	Smoking history (No vs Yes)	0.56	0.31–1.01	0.056
	Treatment strategy (RT alone vs CCRT)	4.63	1.11–19.36	0.036
	Treatment strategy (RT alone vs NAC + CCRT)	4.86	1.17–20.15)	0.029

*Abbreviations*: *RT-PWL* Percentage of weight loss during radiotherapy, *HR* Hazard ratio, *95% CI* 95% confidence interval, *DFS* Disease-free survival, *OS* Overall survival, *DMFS* Distant metastasis-free survival, *LRRFS* Locoregional relapse-free survival, *LDH* Lactate dehydrogenase, *EBV* Epstein-Barr virus

^a^ *P* values were calculated using an adjusted Cox proportional hazards model

### Correlation between RT-PWL_7_ and clinicopathological characteristics

Table 1 presents the correlations between clinicopathological characteristics and RT-PWL_7_. High RT-PWL_7_ patients were more likely to have advanced TNM stage (advanced T, N, and/or overall stage) (*P* < 0.05 for all). With respect to treatment strategy, the proportion receiving RT alone among the low RT-PWL_7_ group was associated with higher receipt compared with high RT-PWL_7_ group (20.2% versus 7.9%; *P* < 0.001). Factors associated with development of high or low RT-PWL_7_ were analyzed. After multivariate analysis, treatment modality, plasma EBV DNA, and N stage remained associated with RT-PWL_7_ (*P* < 0.05 for all; Table 3). Patients treated with RT alone had the lowest risk with development of weight loss during RT. In contrast, CCRT alone or NAC followed by CCRT had a significantly strong correlation with the development of high weight loss (*P* < 0.05 for all). Moreover, patients with advanced N stage (N2-3) were more likely to suffer high weight loss than patients with early N stage (N1-2) (OR, 1.52; 95% CI, 1.12–1.94; *P* = 0.005) during RT.

**Table 3:** Multivariate analysis of prognostic factors for the development of high RT-PWL in patients with NPC

Variable	OR	95% CI for OR	*P* value^a^
T stage			
T1-2	Reference		
T3-4	1.01	0.74–1.38	0.955
N stage			
N0-1	Reference		
N2-3	1.52	1.12–1.94	0.005
LDH, U/L^b^			
< 245	Reference		
≥ 245	1.38	0.83–2.29	0.232
EBV DNA, copy/mL^b^			
< 4000	Reference		
≥ 4000	1.43	1.04–1.96	0.034
Treatment			
RT alone	Reference		
CCRT alone	3.29	2.18–4.96	< 0.001
NAC + CCRT	2.03	1.32–3.13	0.001

*Abbreviations*: *OR* Odds ratio, *95% CI* 95% confidence interval, *LDH* Lactate dehydrogenase, *EBV* Epstein-Barr virus, *RT* Radiotherapy, *CCRT* Concurrent chemoradiotherapy, *NAC + CCRT* Neoadjuvant chemotherapy plus concurrent chemoradiotherapy

^a^ *P* values were calculated using logistic regression models

^b^ All variables were measured before treatment

## Discussion

To our knowledge, this is the longest follow-up analysis for investigating the downward trend in weight loss during treatment among NPC patients. Weight loss was more often observed in the RT period than NAC period. Although the prognostic value of NAC-PWL for NPC was not observed, RT-PWL_7_ ≥ 5% was significantly associated with inferior ten-year DFS, OS, and DMFS for NPC patients. Further analysis revealed that bodyweight remained largely unchanged during RT for the first 2 weeks, and dropped continuously at the following 5 weeks of RT.

Numerous studies [20, 21] have confirmed that weight loss is correlated with poor prognosis among individuals diagnosed with head and neck cancer, including NPC [10, 11]. Results from our study aligned closely with prior findings [10, 11]. There are several potential reasons for these findings. First, critical weight loss may result in loosening of posture fixation, inaccurate radiation field, and significant dosimetric change during RT [22]. Second, reduction in treatment tolerance and radiotherapy breaks could result from weight loss, thus influencing therapeutic efficacy [23, 24]. Third, weight loss is often used as a tool for assessment of newly developed malnutrition, which contributes to weakness in immunity defense mechanism, such as cellular and humoral immunity, phagocyte function, and anatomic barriers. Hence, increasing infection susceptibility and reduced response to malignancy [25, 26]. We must note that the present study failed to confirm the significant impact of RT-PWL_7_ in locoregional control. These finding are reasonable as excellent locoregional control (5-year LRFFS > 90%) is expected for IMRT, therefore actual impact of weight loss on LRRFS would be limited [27, 28].

Previous studies reported that weight loss ≥5% during RT was associated with poor survival [12, 23]. Consistent with previous studies, our results indicated RT-PWL_7_ ≥ 5% was associated with poor DFS, OS, and DMFS. However, Du et al [11] recently assessed weight loss during the entire treatment procedure and observed weight loss ≥10% was an indicator for likelihood of metastasis and overall survival. This inconsistency might be due to some obvious differences between the definition of weight loss in the study by Du et al and the current study. The weight loss during NAC (NAC-PWL) or RT period (RT-PWL) was evaluated separately in our study. However, compared with our study, the weight loss during the entire treatment (including NAC and RT period) was evaluated together by Du et al, which may increase the span of weight loss. Overall, the impact of weight loss on prognosis of NPC can be determined in the present study.

During treatment, numerous factors may influence weight loss among cancer patients [9, 11]. We observed that patients treated with CCRT alone or NAC plus CCRT were more likely to suffer high weight loss during RT when compared with those treated with RT alone. This result was similar with findings by Qiu et al [9] and Du et al [11]. Although encouraging results attained by multimodal therapy for NPC, acute toxicities are more likely to occur during high-intensity chemoradiotherapy [9], including severe oral mucositis, nausea, and vomiting. In the present study, advanced N stage was associated with high weight loss, which was consistent with findings by Du and colleagues. A patient with advanced N stage might receive a higher radiation dose of oropharynx and more aggressive cisplatin-based chemotherapy, potentially exacerbating oropharyngeal pain and oral mucositis. Subsequently, severe oropharyngeal pain and oral mucositis can make eating difficult and lead to weight loss. Other risk factors including radiation technique and segmentation model are partly relevant to oral mucositis and weight loss. Since this research adopts the unified radiation technique and segmentation model, we did not include the above factors for analysis.

Prior studies [10–12] primarily evaluated weight loss at a single time point, usually pre- or post-treatment. For this reason, limited knowledge exists about the dynamic change of weight loss during RT. Since weight loss is common among NPC patients, it is necessary to assess weight change over RT time, potentially providing a more complete understanding on the relationship between bodyweight and survival. Our results indicated that bodyweight remained generally unchanged in the first 2 weeks of RT, and then began to drop relatively stable in the next 5 weeks of RT. The following reasons may explain the observed results. First, the oral mucous membrane reaction of patients is mild, and diet is less affected in the first 2 weeks of RT. With the increasing number of RT, weight loss is gradually accelerated due to oral mucositis, aggravated swallowing pain, and decreased treatment tolerance [4].

Several limitations must be noted. First, we failed to collect pre-existing nutrition status of the patients, which may confound the main findings of this research. Further studies are needed to collect pre-existing nutrition status in nutrition analysis. Second, we lacked detailed information on dietary habit, food intake, and nutritional status. However, during the study period, no standard criteria for nutritional support in patients undergoing RT has been established. Third, due to the lack of date, we couldn’t conduct the analysis of survival outcome against patients who required nutritional support (nasogastric tube feeding or gastrostomy feeding) and those who did not. Fourth, various treatment strategies (i.e., RT alone, CCRT alone, and NAC plus CCRT) may confound the optimal threshold of RT-PWL. Nevertheless, all treatment strategies included in our study were in line with National Comprehensive Cancer Network guidelines. Additionally, the adverse impact of weight loss on prognosis of NPC was still determined in our study. Last but not least, the data used in this study derived from only one institution, where a large proportion of physicians have expertise in diagnosing and treating NPC. Future studies that incorporate external validation are needed.

## Conclusions

In conclusion, the downward trend of weight loss at every week during RT was outlined, and, after the end of RT, the optimal threshold for RT-PWL adversely impacting NPC prognosis was 5%. Further research is needed on limiting weight loss during RT under 5% in clinical practice as a result of the detrimental impact of RT-PWL_7_ ≥ 5% on survival outcomes. Additionally, treatment strategy, plasma EBV DNA, and N stage were associated with weight loss. These findings would be helpful in selecting patients for preventive measures before RT.

## Data Availability

The datasets analyzed during the current study are available in the Research Data Deposit (RDD) public platform www.researchdata.org.cn, with the approval RDD number of RDDA2019001296. The raw dataset is available from the corresponding author upon reasonable request.

## Abbreviations

NPC: Nasopharyngeal carcinoma
RT: Radiotherapy
KPS: Karnosfky performance score
IMRT: Intensity-modulated radiotherapy
EBV: Epstein-Barr virus
AJCC: American Joint Commission on Cancer
PTV: Planning target volume
GTVnx: Nasopharyngeal gross tumor volume
GTVnd: GTV of the metastatic lymph nodes
CTV1: High-risk clinical target volume
CTV2: Low-risk clinical target volume
NAC: Neoadjuvant chemotherapy
CCRT: Concurrent chemoradiotherapy
LDH: Lactate dehydrogenase
hs-CRP: High sensitivity C-reactive protein
WPre-RT: Bodyweight before RT
NAC-PWL: Percentage of weight loss during neoadjuvant chemotherapy
RT-PWL: Percentage of weight loss during radiotherapy
RT-PWL_7_: Percentage of weight loss at week 7 of radiotherapy
DFS: Disease-free survival
DMFS: Distant metastasis-free survival
OS: Overall survival
LRRFS: Locoregional relapse-free survival
RPAs: Recursive partitioning analyses
